# Dyslexia and Developmental Language Disorder: comorbid disorders with distinct effects on reading comprehension

**DOI:** 10.1111/jcpp.13140

**Published:** 2019-10-20

**Authors:** Margaret J. Snowling, Marianna E. Hayiou‐Thomas, Hannah M. Nash, Charles Hulme

**Affiliations:** ^1^ Department of Experimental Psychology University of Oxford Oxford UK; ^2^ Department of Psychology University of York York UK; ^3^ Department of Psychology University of Leeds Leeds UK; ^4^ Department of Education University of Oxford Oxford UK

**Keywords:** Reading comprehension, dyslexia, Developmental Language Disorder, decoding, language

## Abstract

**Background:**

Reading comprehension draws on both decoding and linguistic comprehension, and poor reading comprehension can be the consequence of a deficit in either of these skills.

**Methods:**

Using outcome data from the longitudinal Wellcome Language and Reading Project, we identified three groups of children at age 8 years: children with dyslexia (*N* = 21) who had deficits in decoding but not oral language, children with Developmental Language Disorder (DLD; *N* = 38) whose decoding skills were in the normal range, and children who met criteria for both dyslexia and DLD (*N* = 29).

**Results:**

All three groups had reading comprehension difficulties at the ages of 8 and 9 years relative to TD controls though those of the children with dyslexia were mild (relative to TD controls, *d* = 0.51 at age 8, *d* = 0.60 at age 8); while the most severe problems were found in the comorbid dyslexia + DLD group (*d* = 1.79 at age 8, *d* = 2.06 at age 9) those with DLD also had significant difficulties (*d* = 1.56 at age 8, *d* = 1.56 at age 9).

**Conclusions:**

These findings confirm that children with dyslexia or DLD are at‐risk for reading comprehension difficulties but for different reasons, because of weak decoding in the case of dyslexia or weak oral language skills in the case of DLD. Different forms of intervention are required for these groups of children, targeted to their particular area(s) of weakness.

## Introduction

It is well established that oral language is the foundation of learning to read (Storch & Whitehurst, 2002; Hulme et al., 2015; Lervag, Hulme & Melby‐Lervag, 2018) and that children with a history of oral language difficulties are at high risk of reading problems (Catts, Fey, Tomblin & Zhang, 2002; Snowling, Bishop & Stothard, 2000). Furthermore, the nature of the reading problem differs according to the language profile of the child: Phonological deficits are strongly associated with poor decoding while problems with vocabulary, grammar, and receptive language are more strongly associated with reading comprehension difficulties (Snowling & Hulme, 2012). Notwithstanding this, it is important to recognize that many children have both decoding and reading comprehension problems (Catts, Adlof, Hogan & Weismer, 2005; Language and Reading Consortium, 2015).

Turning to the relationship between neurodevelopmental disorders of language and reading, Bishop and Snowling (2004) argued that it is important to consider two dimensions of variation: Individual differences in phonological and broader oral language skills. Drawing upon the Simple View of Reading, that reading comprehension is the product of decoding and linguistic comprehension (Gough & Tunmer, 1986), this two‐dimensional (2D) model proposes that dyslexia is associated with poor phonological skills and hence poor decoding, while Developmental Language Disorder (also referred to as specific language impairment) is associated with poor reading comprehension. In turn, Developmental Language Disorder (DLD) can co‐occur with poor phonology (and hence dyslexia) or with proficient phonological skills (the poor comprehender profile, Nation et al., 2004). Moreover, the developmental course of language difficulties is important: Children whose language difficulties resolve by the time of formal reading instruction are less likely to go on to experience decoding difficulties than those whose language difficulties persist into the school years (Bishop & Adams, 1990; Catts, et al., 2005; Stothard, Snowling, Bishop, Chipchase & Kaplan, 1998). Subsequent studies have broadly confirmed that dyslexia and DLD are separate disorders but comorbidity between them is common (Bishop, McDonald, Bird, & Hayiou‐Thomas, 2009; Ramus, Marshall, Rosen, and van der Lely, 2013; Snowling, Nash, Gooch, Hayiou‐Thomas & Hulme, 2019).

Here, we present data from the final phases of the project in which we followed the literacy development of a high‐risk sample of children from the age of 3½ years, recruited to the study from one of three groups: children with a family history of dyslexia, children with preschool language impairment, and typical controls (Nash, Hulme, Gooch & Snowling, 2013). We focus on the reading comprehension outcomes of these children at the ages of 8 and 9 years (*t5 , t6*) and consider these in two ways. First, we examine the performance of children recruited in preschool as ‘at‐risk’ of dyslexia (those at family risk and those with preschool language difficulties) compared with typical (low‐risk) controls. In terms of group differences in reading comprehension, we expect children at family risk of dyslexia to show poorer performance than low‐risk controls (Snowling & Melby‐Lervåg 2016) but for them to be less impaired than children who had preschool language problems (because language skills are the foundation of both decoding‐related phonological skills and comprehension processes; Hulme et al., 2015). However, we expect outcomes to be moderated in each of the groups by language skills around the time of school entry. We therefore also report data according to language status at age 5½ years (*t3*).

Second, we examine the reading comprehension abilities of children classified at age 8 into three diagnostic groups according to their language and reading attainments: dyslexia, Developmental Language Disorder (DLD) and comorbid dyslexia + DLD. A retrospective analysis of data collected in earlier test phases (Snowling et al., 2019) showed that children with dyslexia had relatively specific difficulties with phonology from as early as the preschool period, whereas children with DLD showed a wide range of language impairments from preschool onwards. Children with DLD‐only had milder phonological difficulties than the other two groups and these appeared to resolve over time. For children with dyslexia + DLD, difficulties with decoding and phonology were generally more severe than those observed in dyslexia or DLD without dyslexia. Within the framework of the Simple View of Reading, children in each of the ‘diagnostic’ groups are predicted to have problems with reading comprehension but for different reasons: For children with dyslexia, decoding is expected to be the prime obstacle to comprehension; for children with DLD, reading comprehension will be compromised by weaknesses in broader language skills (Nation, Clarke, Marshall & Durand, 2004; Nation, Cocksey, Taylor & Bishop, 2010). To assess this hypothesis, we used nonword reading as a relatively pure measure of decoding skill and vocabulary knowledge as a measure of language because of its strong correlation with language comprehension. Importantly, however, when dyslexia and DLD co‐occur, we expect more severe reading comprehension difficulties, reflecting problems with both decoding and language comprehension (and top‐down use of context will be compromised, Nation & Snowling, 1998).

A further consideration is timing: Recent studies suggest that the relationship between the two skills which underlie reading comprehension (decoding and language) changes over time, with decoding accounting for more variance early in reading development and language being the stronger predictor of reading comprehension later when decoding becomes automatized (Castles, Rastle & Nation, 2018; Lervag, Hulme, & Melby‐Lervag, 2018; Vellutino, Tunmer, Jaccard & Chen, 2007). It can therefore be hypothesized that the reading comprehension difficulties of children with dyslexia may decrease over time, while those of the children with DLD (who do not have dyslexia) may increase. Although intact reading comprehension has been posited in dyslexia (Frith & Snowling, 1983; Nation & Snowling, 1998), this has seldom been evaluated in a longitudinal study; we were interested in investigating its severity.

## Method

Data are reported from the final phases of the Wellcome Language and Reading Project (*t5, t6*). *Ethical considerations*: Clearance for the study was provided by the University of York, Department of Psychology Ethics Committee and the NHS Research Ethics Committee. Parents provided informed consent for their child to participate. Children were assessed by trained testers (see Snowling et al., 2019 for details).

### Participants

Families were recruited to the study via speech and language therapy services and via advertisements placed in local newspapers, nurseries and the webpages of support agencies for children with reading and language difficulties. Following recruitment when children were 3½ years, 260 children were classified using a two‐stage process to determine whether they were at family risk of dyslexia (FR) and then to ascertain whether they had a preschool language impairment (LI) placing them at‐risk of Developmental Language Disorder. Seventy‐one children were recruited as controls and had no history of language problems or other risk factors (for details see Nash et al., 2013). There was a small amount of attrition; data from all children who remained in the sample at *t*5 (*N* = 234) and at *t6* (*N* = 224) are included in the present analyses (see Figure S1 in the Supporting Information for Participant Flow). At *t5*, the mean age of the sample was 96.73 months (*SD* = 5.92) and at *t6*, 109.72 months (*SD* = 6.18).

#### Classification of Outcomes at age 8 (*t5*)

Reading and language skills are continuously distributed in the population and there is no clear cutoff between ‘typical’ and ‘impaired’ levels of performance. However, when considering whether an *individual* is functionally impaired, or requires intervention, it is important to agree a cutoff criterion. In this project, we were interested in the role of two developmental risk factors as predictors of dyslexia outcome (family risk of dyslexia and preschool language difficulties); it follows that decisions regarding how best to define binary outcomes on continuous scales had to be made. The criteria adopted reflected our aim to assess the predictors of dyslexia in 'at‐risk' groups of individuals. Previous family‐risk studies have used variable criteria to compare dyslexia and normal reading, with < 10th centile being a common cutoff for dyslexia (Snowling & Melby‐Lervåg 2016); since 'dyslexia' was the primary outcome measure here we decided to use a criterion at *8 *years of −1.5*SD* (equivalent to < 7th centile) in reading and spelling. A composite score was formed by averaging the standardized (norm‐referenced) scores from the Single Word Reading Test (*SWRT* 6–16, Foster, 2007) and the Wechsler Individual Achievement Test, Spelling Test (*WIAT II*; Wechsler, 2005). The mean of this composite standard score for the TD sample was 106.88 (*SD* = 11.68). Dyslexia was defined as falling at least 1.5 standard deviations below the mean of the TD group (a score of 89 or less). Of the 234 children remaining in the sample, 50 were identified as dyslexic according to this criterion, 184 were normal readers.

At the time of recruitment, the language‐impaired group was selected as falling −1*SD* below the mean (a standard score of < 86 according to test norms) or below criterion on 2/4 tests of receptive and expressive language (Nash et al., 2013). It was important that similar criteria be used at outcome. Developmental Language Disorder (DLD) at was defined by performance on a composite score formed by averaging the standardized (*z*) scores on three tests: *Expressive Vocabulary* (*CELF‐4, UK* – Wiig, Secord, & Semel, 2006), *Test for Reception of Grammar* (*TROG‐II* – Bishop, 2003) and *Recalling Sentences* (*CELF 4*) rather than by the method of ‘diagnosis’ based on below average performance on 2/4 tests. The mean of the composite (*z*) language score for the TD sample was 108.1 (*SD* = 8.7); DLD was defined by a *z* score of 85 or below on this measure. Using this criterion, 67 children were classified as reaching criterion for DLD, 167 as having normal language. It should be noted that while this is more than two standard deviations below the TD mean, Snowling and et al., (2016) justified the method by showing it classified individuals into categories with similar inclusions as the method of falling below −1*SD* on two tests.

Grouping the children according to whether they had pure or comorbid disorders yielded three groups at age 8 years (*t5*): 21 children were classified as fulfilling diagnostic criteria for dyslexia (14M:7F); 38 for DLD (23M:15F); and 29 for dyslexia + DLD (22M:7F; Snowling et al., 2019). At age 9 years*,* the numbers remaining in each group were as follows*:* 20 dyslexia; 38 DLD; 23 dyslexia + DLD; and 64 TD control. In the sample as a whole, 146 children (77M: 69F) had a good outcome (neither dyslexia nor DLD). Of those who had been recruited as typically developing at *t1* (with neither a risk of DLD nor reading difficulties), 64 (out of 71) remained in the sample and had a ‘normal’ reading and language outcome. Here, they are used as a comparison group (TD control) against which to assess the size of deficits in the clinical groups. Data from these groups on the tests used for classification are given in Table S1. For key variables, Table S2 provides data for children with a ‘normal outcome’ who were recruited to at‐risk groups at *t1* (‘at‐risk normal’ *N* = 82).

### Tests and procedures

Each child was administered a large battery of tests in a 2‐hr session. The tasks are described fully elsewhere (Snowling et al., 2019), and details of the language measures used to classify the children recruited at *t1* (age 3½) are given in Appendix S1 (Nash et al., 2013 for more details); here, the focus is on the tests used to classify the children into diagnostic groups and on language, reading, and reading comprehension skills. Measures of reliability are based on those in test manuals for standardized tests and for the current sample on non‐standard measures given at 8 years. In addition, parents and teachers completed questionnaires at the time of each assessment. These comprised ratings of behaviour, attention, motor skills and communication but were not used in the current study. Parents also provided information about the child’s interest in reading and related activities.

#### Language

##### Receptive Grammar (*t5*)

The *Test for the Reception of Grammar* (*TROG‐II*: Bishop, 2003) was administered (α = .88). The child heard sentences of increasingly complex syntactic structure and had to select from a choice of four pictures the one that conveyed the meaning of each (80 items maximum).

##### Expressive Grammar (*t5*)

The *CELF‐4 Recalling Sentences* (Wiig et al., 2006) was administered (α = .92). Children repeat verbatim a list of sentences which increase in length and grammatical complexity (32 items maximum).

##### Morphological Inflection (*t5*)

*CELF‐4 Formulated Sentences* (α = .76) measured expressive grammar. The child was shown a picture and given a word to use in a sentence describing the picture (28 items maximum).

##### Vocabulary (*t5*, *t6*)

Vocabulary knowledge was measured by two tests at each time: The *CELF 4 Expressive Vocabulary* test (27 words) (Wiig, et al., 2006) including 8 extension items (α = .66) and the *Receptive One Word Picture Vocabulary Test (170 words maximum) (ROWPVT; *Brownell, 2000) (α = .95). In the CELF 4 expressive vocabulary test the child was asked to name an object or an action from a picture, whereas in the ROWPVT the child heard a word and had to select the picture that shows the meaning of the word.

#### Reading

Children were given a battery of reading tests tapping word reading, nonword reading, and reading comprehension skills.

##### Word Reading (*t5*, *t6*)

To assess single word reading at *t5*, the *Single word reading test* (S*WRT;* Foster, 2007) (60 words, α = .98) was given. The Exception Words from the *Diagnostic Test of Word Reading Processes* (Forum for Research in Language and Literacy, 2012) was given at *t5* and *t6* (30 words, α = .97). To assess reading fluency for words, the Test of Word Reading Efficiency (TOWRE; Torgersen, Rashotte & Wagner, 1999) which requires the rapid reading of a list of 104 words was given (*t5*, *t6*) (test–retest reliability = .93).

##### Nonword Reading Skill (*t5*, *t6*)

To provide a robust measure of decoding skills, nonword reading was measured. At *t5*, the Graded Nonword Reading test comprising 20 nonwords was given (Snowling et al., 1996) (α = .78) and at *t6*, the nonwords from the *Diagnostic Test of Word Reading Processes* (Forum for Research in Language and Literacy, 2012) (30 nonwords, α = .96); to assess nonword reading fluency, the Test of Word Reading Efficiency (TOWRE; Torgersen, Rashotte & Wagner, 1999) which requires the rapid reading of a list of 63 nonwords was given (*t5*, *t6*) (test–retest reliability = .93).

##### Reading Comprehension (*t5*, *t6*)

The child read passages from the *York Assessment of Reading for Comprehension (YARC Passage Reading;* Snowling et al., 2009), and accuracy was monitored. The child then answered 8 spoken comprehension questions about each passage. Comprehension ability scores are calculated based on the two most difficult passages the child read (average α = .63).

## Results

Table 1 shows the reading comprehension skills of the children in the sample, grouped according to risk status from *t1* (3½ years). We use analyses of variance to assess group effects with Bonferroni tests for subgroup comparisons; we also report effect sizes and 95% confidence intervals since sample sizes are relatively small for the subgroups.

**Table 1:** Reading comprehension skills measured at age 8 and age 9 for the risk groups compared with TD controls (mean, *SD*)

	TD control		Dyslexia risk (FR)		Preschool language difficulties (LI)		FR + Preschool language difficulties	
	Mean	*SD*	Mean	*SD*	Mean	*SD*	Mean	*SD*
Read Comp^a^ *t5*	60.58	8.71	57.26	9.28	51.41	7.05	49.07	7.37
Read Comp^a^ *t6*	66.94	7.56	63.65	7.56	54.82	12.67	52.88	9.01

a York Assessment of Reading and Comprehension (YARC), ability score.

There was an overall group difference at *8 *years* (F*(3,210 = 16.32, *p *< .001); however, while the TD controls had marginally higher reading comprehension scores than those at family risk of dyslexia (FR), the difference was not statistically significant. Further, both the TD and FR groups performed better than the two groups who experienced preschool language difficulties (at the time of the study, we described these as language impaired (LI) and language impaired combined with family risk of dyslexia (FRLI)). This pattern was replicated at 9 years* (F*(3,199) = 24.65, *p *< .001).

A more critical issue relates to timing. It was predicted that the status of the child’s language system at 5½ years would predict later reading skill. To explore this issue, we used the classification of children into groups whose language difficulties had resolved (*N* = 12), persisted (*N* = 39) or emerged (*N* = 18) at *t3*, as reported by Snowling et al., (2016). Using the reading comprehension outcome of the TD control group as a benchmark, the group who had resolved their language difficulties did not differ significantly from controls at 8 years (*d *= .39) but the groups who had either emerging or persisting language difficulties performed much more poorly (*d*s = 1.43 and 1.62 respectively). At 9 years*,* the pattern of performance of the groups was similar with one exception: Those whose language difficulties had resolved by 5½ years did now show a deficit, albeit mild (*d *= .4) and not very different from that the previous year. Moreover, it is noteworthy that there was considerable variation in this group (mean ability score = 58.0, *SD* = 17.13), and given the small sample size, this effect needs to be interpreted with caution.

Table 2 shows the performance of the three outcome groups (dyslexia, DLD and dyslexia + DLD) and the TD control group on the individual language, reading and nonword reading measures at ages 8 and 9 (*t5, t6*). It is clear that the pattern in the data is as expected given the way in which the groups were classified. However, a few points are worth noting. First, although the group with DLD do not fulfill criteria for dyslexia, their reading skills are less good than those of the TD controls (*d* = 1.10 for exception word reading). Second, although the group with dyslexia performed within the normal range across language measures, they scored much less well than the group with DLD on the word and nonword reading measures: Group differences were particularly marked on nonword reading where the dyslexia deficit was large (*d*s = 2.13–2.29), whereas it was the smallest deficit for the DLD group (*d*s = .59–.68).

**Table 2:** Decoding, vocabulary, and reading comprehension by outcome group at age 8 and age 9 (dyslexia, DLD, comorbid dyslexia + DLD, and TD control group) showing effect size of deficit between each clinical group and TD control (Cohen’s *d*; 95% confidence intervals)

	TD control		Dyslexia			DLD			Dys + DLD		
	Mean	*SD*	Mean	*SD*	*d*	Mean	*SD*	*d*	Mean	*SD*	*d*
Vocab^a^ *t5*	0.52	0.63	0.13	0.59	0.63 [0.12; 1.13]	−0.86	0.52	2.33 [1.81; 2.84]	−1.06	0.63	2.51 [1.93; 3.07]
Vocab^a^ *t6*	0.46	0.77	0.10	0.77	0.77 [0.26; 1.27]	−1.78	0.56	3.60 [2.96; 4.24]	−0.97	0.60	3.29 [2.63; 3.93]
Nonword Reading^b^ *t5*	0.44	0.59	−1.10	0.70	2.47 [1.85; 3.09]	0.00	0.64	0.72 [.31; 1.14]	−1.29	0.76	2.68 [2.08; 3.27]
Nonword Reading^b^ *t6*	0.44	0.66	−1.10	0.69	2.33 [1.69; 2.97]	−0.06	0.66	0.76 [.33; 1.19]	−1.32	0.81	2.50 [1.92; 3.07]
Read Comp^c^ *t5*	60.98	8.48	56.45	9.85	0.51 [0.03; 0.99]	48.84	6.44	1.56 [1.10; 2.01]	45.96	8.10	1.79 [1.24; 2.34]
Read Comp^c^ *t6*	67.38	7.13	63.18	6.70	0.60 [0.05; 1.14]	55.74	8.05	1.56 [1.09; 2.03]	51.39	9.39	2.06 [1.46; 2.62]

a Vocabulary factor score (expressive and receptive vocabulary).

b Nonword reading factor score (nonword reading accuracy and timed nonword reading efficiency).

c York Assessment of Reading and Comprehension (YARC), ability score.

To provide reliable measures of nonword reading (as a marker of decoding) and vocabulary (as a marker of language) skills at 8 years and *t6*, we used principal component analysis to derive factor scores. Correlations among the two measures of nonword reading were high (*r* = .80 at 8 years, *r* = .86 at 9 years); a nonword reading factor score was derived from these two measures (at 8 years, there were high loadings of .85 for each on a single factor, eigenvalue = 1.43; at 9 years, the loadings were .89, eigenvalue = 1.59). Correlations among the two measures of vocabulary were moderate (*r* = .68 at 8 years, *r* = .60 at 9 years); a vocabulary factor score was derived from these two measures (at 8 years, there were high loadings of .76 for each on a single factor, eigenvalue = 1.15; at 9 years, the loadings were .70, eigenvalue = .99). Although different from the composite language score used to classify the groups at 8 years when additional tests were used, the vocabulary factor correlated highly with it (*r *= .89).

Table 3 shows the factor scores for the four groups at 8 years and 9 years together with ability scores for reading comprehension. Effect sizes are given for the differences between each of these groups and the TD control group (children recruited with neither a risk of DLD nor reading difficulties who had a ‘normal’ outcome at age 8 years). At both 8 years and 9 years, the overall group effect on vocabulary was significant (*t5: F*(3,233) = 84.12, *p *< .001; *t6*: *F*(3,223) = 52.06, *p *< .001). At neither time was there a significant difference between the performance of the typically developing group and the group with dyslexia while both of these groups differed from the DLD and comorbid groups (who performed at the same level to each other). Notwithstanding this, 8/21 (38%) of the group with dyslexia showed weak vocabulary (below 1*SD* of the TD mean) at 8 years (28% at 9 years), compared with 97% of the DLD and 90% of the comorbid group (79 and 86% at 9 years). Turning to nonword reading, there were significant group differences at both time points (*t5: F*(3,231) = 77.77*, p *< .001; *t6: F*(3,223) = 72.77, *p *< .001) with the TD group performing better than all of the clinical groups. The DLD group performed significantly better than the dyslexia group at 8 years in nonword reading, consistent with their ‘diagnosis’ and the group with dyslexia and comorbid dyslexia + DLD groups performed at the same level as each other at both times.

**Table 3:** Mean (*SD*) reading and vocabulary skills at age 8 (*t5*) and age 9 (*t6*) for dyslexia, DLD, and comorbid group relative to TD control (normal outcome) with standardized mean differences (Cohen’s *d*) between the clinical groups and control group

	TD control		Dyslexia			DLD			Dys + DLD		
	Mean	*SD*	Mean	*SD*	*d*	Mean	*SD*	*d*	Mean	*SD*	*d*
Expressive vocab^a^ *t5*	50.02	6.26	45.90	4.46	0.70 [0.19; 1.2]	34.24	5.99	2.56 [2.02; 3.09]	31.72	7.17	2.79 [2.19; 3.39]
Expressive vocab^a^ *t6*	53.25	5.42	50.28	7.94	0.49 [−0.04; 1.02]	41.09	6.02	2.16 [1.64; 2.67]	37.50	7.72	2.54 [1.96; 3.11]
Receptive vocab^b^ *t5*	102.63	12.36	96.81	13.35	0.46 [−0.04; 0.96]	84.05	11.33	1.55 [1.09; 2.00]	81.45	11.16	1.76 [1.25; 2.27]
Receptive vocab^b^ *t6*	113.14	14.16	106.44	12.38	0.49 [−0.04; 1.01]	91.47	9.94	1.68 [1.20; 2.16]	88.71	10.21	1.86 [1.34; 2.38]
NW reading^c^ *t5*	17.06	2.92	9.00	5.09	2.26 [1.66; 2; 86]	15.16	3.63	0.59 [0.18; 1.00]	7.00	5.51	2.58 [2.0; 3.15]
NW reading^d^ *t6*	23.58	5.00	11.78	5.64	2.29 [1.66; 2.92]	20.09	5.34	0.68 [0.25; 1.11]	9.96	6.81	2.43 [1.86; 2.99]
Exception words^d^ *t5*	23.57	3.56	12.05	5.96	2.69 [2.03; 3.33]	19.18	4.60	1.10 [0.66; 1.53]	9.93	6.30	2.99 [2.35; 3.62]
Exception words^d^ *t6*	25.67	2.66	17.78	6.56	2.06 [1.44; 2.66]	22.32	3.66	1.10 [0.65; 1.54]	13.32	6.39	2.98 [2.35; 3.59]
Wd Read Rate^e^ *t5*	65.09	10.06	36.57	15.16	2.48 [1.86; 3.10]	55.26	11.79	0.92 [0.49; 1.33]	33.11	18.05	2.46 [1.89; 3.03]
NW Read Rate^e^ *t5*	35.33	10.43	13.29	7.58	2.24 [1.64; 24]	27.87	10.24	0.72 [0.30; 1.13]	11.78	8.44	2.38 [1.81; 2.95]
Wd Read Rate^e^ *t6*	70.97	8.51	49.11	15.88	2.08 [1.46; 2.69]	62.85	10.18	0.89 [0.45; 1.32]	39.43	19.98	2.42 [1.84; 2.98]
NW Read Rate^e^ *t6*	40.00	10.16	18.22	10.44	2.13 [1.51; 2.74]	32.26	10.96	0.74 [0.31; 1.17]	15.36	11.70	2.31 [1.75; 2.87]

a Clinical Evaluation of Language Fundamentals (CELF4).

b Receptive One Word Vocabulary Test ROWPVT.

c Graded Nonword Reading Test (GNWRT).

d Diagnostic Test of Word Reading Processes (DTWRP).

e Test of Word Reading Efficiency (TOWRE).

Turning to the key outcome measure of reading comprehension, as predicted, the DLD group performed less well than the group with dyslexia but, contrary to prediction, the performance of the group with comorbid dyslexia + DLD was only marginally, and not significantly, worse than the group with DLD. Similarly, while the group with dyslexia performed less well than the TD controls in reading comprehension (*d*s = .51 at *t5*, .60 at *t6*), the group differences were not statistically significant. Table S2 includes data for the group of children with a ‘normal outcome’ who belonged to an ‘at‐risk’ group at *t1* for comparison purposes: the ‘at‐risk normal outcome’ group performed similarly to the TD control group on all outcome variables, with effect sizes ranging from *d *= .10 to .40; importantly, the at‐risk ‘normal outcome’ group performed better than the dyslexia group across all measures (*r*s approx. .4) except for one at 9 years when their reading comprehension skills were similar to those of the children with dyslexia).

**Figure 1:**
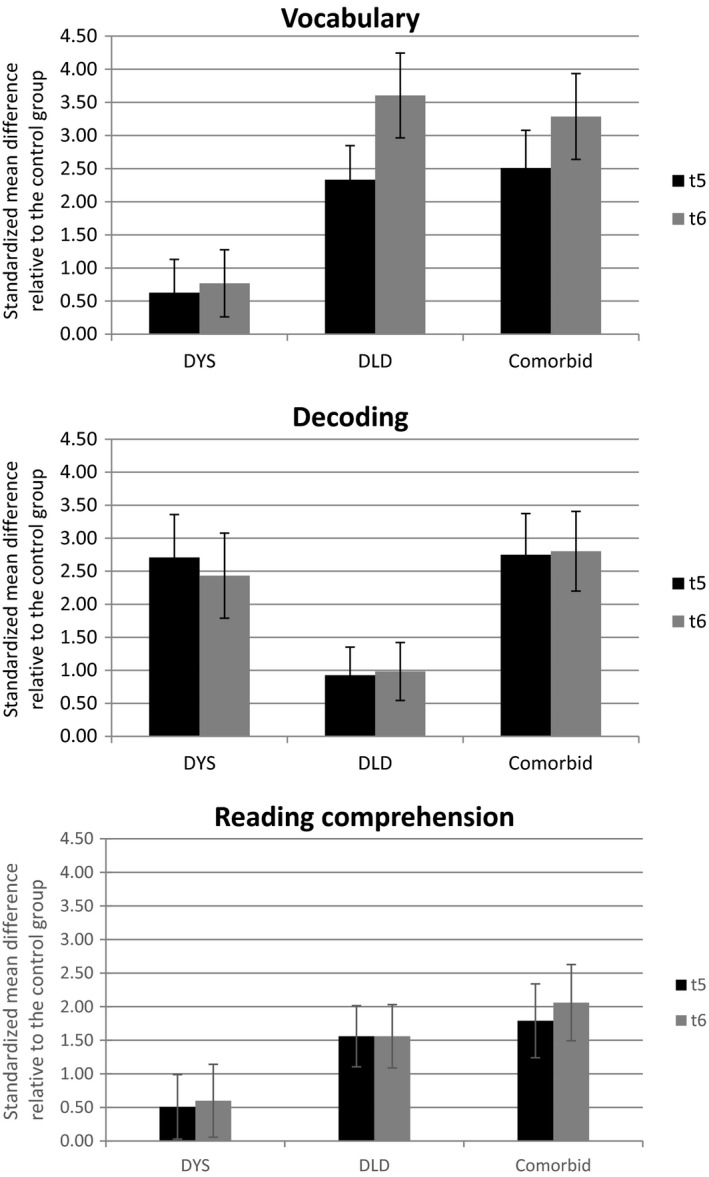
Standardized mean differences for the dyslexia, DLD and comorbid dyslexia + DLD groups relative to the TD control group at age 8 (*t5*, dark) and age 9 (*t6*, light). Error bars are 95% CIs: upper panel, Vocabulary; middle panel, Decoding; and lower panel, Reading Comprehension skills

Figure 1 shows the data from the vocabulary, nonword reading, and reading comprehension measures plotted in terms of the effect size of the deficit relative to TD controls. The height of each bar represents the size of the deficit for that group relative to the control group.

Relative to TD controls, the group with dyslexia show mild deficits in vocabulary at age 8 (*t5*) and 9 (*t6*) (upper panel) but these skills are within the ‘normal range’ and the deficit is much smaller than that shown by the two DLD groups. For nonword reading, our measure of decoding (middle panel), the deficits observed in the groups with dyslexia and dyslexia + DLD are large at both time points and do not differ. The group with DLD also has significant deficits relative to controls but they are much less seriously affected than the other two groups. The lower panel shows the reading comprehension data. The two groups with DLD show deficits in reading comprehension whereas the performance of the group with dyslexia is within the normal range. There is no evidence of any significant change in the pattern of performance of any of the groups over time.

Finally, we assessed the hypothesis that the reading comprehension impairment in dyslexia + DLD reflects the additive combination of deficits associated with dyslexia and with DLD. The group with dyslexia + DLD have a larger deficit than the other two groups at both time points (*d* = 1.79 at age 8, *d* = 2.06 at age 9); however, it is only at 9 years that it approximates that of the additive combination of deficits in dyslexia (*d *= .51 at age 8, *d *= .60 at age 9) and in DLD (*d* = 1.56 at age 8, *d* = 1.56 at age 9).

## Discussion

We have examined the reading comprehension outcomes at age 8‐9 years of a large group of children recruited in preschool for being at family risk of dyslexia or for having language difficulties and a comparison group of children with typical development. At age 8 years, the children were classified into 3 groups: dyslexia, DLD, or comorbid dyslexia + DLD. The rate of comorbidity between DLD and dyslexia is extremely high in this ‘at‐risk’ sample; about 48% of children diagnosed as having DLD also fulfill the criteria for the diagnosis of dyslexia and 58% of those classified as dyslexic have DLD (Snowling et al., 2019). As we predicted all three clinical groups have reading comprehension difficulties though these are mild in the group with dyslexia and more severe in the two DLD groups. We also examined outcomes in terms of preschool risks. Generally, children at family risk of dyslexia performed within the normal range for reading comprehension while preschool language difficulties were predictive of later reading comprehension impairment. However, reading comprehension outcome depended on whether or not the child had a concurrent language difficulty. Indeed, we showed that children whose language disorder had resolved by age 5½ years were less likely to experience difficulties than those whose problems were persistent or had emerged around school entry, in line with the ‘critical age’ hypothesis of Bishop and Adams (1990).

From a theoretical perspective, our findings align well with predictions from the Simple View of Reading (Gough & Tunmer, 1986). According to this view, reading comprehension is the product of decoding and language comprehension ability and deficits in either skill will inevitably lead to reading comprehension difficulties. In the current study we have shown that subgroups of children can be identified with decoding difficulties alone, (‘pure dyslexia’), with language disorder in the absence of decoding difficulties (‘pure DLD’), and with both deficits (comorbid dyslexia + DLD)(Snowling et al., 2019). These three groups all show reading comprehension impairments, but for different reasons, and with differing severity, as predicted by the Simple View of Reading.

The children with ‘pure’ dyslexia in our sample have relatively good oral language skills in the face of significant decoding deficits (as indexed by poor nonword reading) and show only mild deficits in reading comprehension. It follows that reading comprehension difficulties are likely a reflection of problems in decoding text (although it may be relevant that, despite their generally adequate levels of language, about a third of the sample had lower levels of vocabulary which could be expected to compromise comprehension to some extent).

In contrast to children with dyslexia, we identified a group of children with relatively pure DLD whose decoding skills were in the normal range for their age. Again, in line with the Simple View of Reading, these children’s reading comprehension difficulties reflect the fact that their language comprehension abilities are not sufficient for them to comprehend the texts that they can decode (cf. Bishop, McDonald, Bird & Hayiou‐Thomas, 2009). Among this group, roughly one half (55%) had reading comprehension skills one standard deviation below their single word reading skill and hence might be classified as ‘poor comprehenders’ (Nation et al., 2004). Finally, as expected, the group of children identified with dyslexia + DLD showed the most severe reading comprehension problems given their dual deficit in decoding and language skills. However, the size of the deficit at 8 years is less marked than we expected, while arguably, at 9 years*,* the size of their deficit reflects the additive combination of problems of decoding and language comprehension; in the face of deficits in both of the main processes underpinning reading comprehension, compensation does not seem possible for this group.

Together the findings underline the dissociation between dyslexia and DLD. We know from our longitudinal study that children with dyslexia experience phonological processing problems from preschool onwards and these compromise learning to read; here, we show that these learning problems manifest themselves principally as decoding rather than reading comprehension problems, in line with the findings from other studies of children at family risk of dyslexia (Snowling & Melby‐Lervåg 2016). In contrast, the primary deficit in children with DLD appears to be in broader language skills that compromise reading comprehension even when decoding is intact. However, in line with the two‐dimensional view of Bishop and Snowling (2004), the DLD profile can be found in pure form (as sometimes observed in ‘poor comprehenders’) or comorbid with dyslexia. Our earlier finding that pure DLD and DLD with dyslexia have different developmental courses, together with the current findings leads us to speculate that dyslexia and DLD do not simply differ in severity but are separable conditions, possibly with different etiologies.

In this study, reading comprehension was assessed using only a single task in which comprehension was assessed verbally. It is probable that children with dyslexia would have more significant problems if reading comprehension was measured by a test in which questions have to be read and/or answered in writing (Keenan, Betjemann & Olson, 2008). The sample in the present study was selected from an ‘at‐risk’ population, and given the relatively small sample size, there is need for caution regarding the generalization of the results. Moreover, clinical groups were formed based on arbitrary cutoffs on dimensions of reading and language and it was notable that, even when between group differences were not significant, scrutiny of effect sizes suggested a more continuous distribution of impairment (for example, the DLD‐only group was somewhat impaired in reading, especially of exception words; and the group with dyslexia showed mild vocabulary weaknesses).

Nevertheless, from an educational perspective, one striking finding is the high rate of reading comprehension and decoding problems found in our ‘at‐risk’ sample. From the 161 children recruited at age 3½ years for being at family risk of dyslexia, or for having a preschool language impairment, who remained in the sample at, 88 (55%) had clinically significant reading or language problems at age 8 years (dyslexia, DLD, or both), It is clear from these figures that a family history of reading problems, or preschool language problems, place children at substantial risk of later reading and language difficulties. Although the present study involved only case–control comparisons and cannot therefore confirm causal hypotheses, theoretically, the findings align with other recent work showing that early language skills appear to be critical in providing the foundations for the development of decoding skills as well as having direct effects on the development of reading comprehension skills (Hjetland, Brinchmann, Scherer, & Melby‐Lervåg, M., 2017; Hulme et al., 2015; Lervåg, Hulme & Melby‐Lervåg, 2018). Our findings also have important implications for educational practice and suggest that for children at‐risk of reading problems the early language profile they show should inform pathways for intervention. Furthermore, language interventions may be particularly beneficial (e.g., Fricke, Bowyer‐Crane, Haley, Hulme, & Snowling, 2013; Hagen, Melby‐Lervåg & Lervåg, 2017; Rogde, Melby‐Lervåg, & Lervåg, 2016) and multicomponential approaches should also be considered (Clarke, Snowling, Truelove & Hulme, 2010; Kendeou, van den Broek, Helder & Karlesson, 2014).

## Supporting information


**Appendix S1.** Measures used to classify groups at *t1*.
**Figure S1.** Participant flow through Wellcome Language and Reading Study.
**Table S1.** Language and literacy performance on tests used to classify outcomes at 8 years (Standard Scores).
**Table S2.** Performance of the at‐risk normal outcome group relative to TD control and dyslexia group at *t5*.
