# Supplementary material for: Dyslexia and Developmental Language Disorder: comorbid disorders with distinct effects on reading comprehension
Source: J Child Psychol Psychiatry. 2019 Oct 20;61(6):672–80. doi: 10.1111/jcpp.13140 (PMC7317952; doi:10.1111/jcpp.13140)
Supplement: Supplementary file 1 — Appendix S1. Measures used to classify groups at t1. Figure S1. Participant flow through Wellcome Language and Reading Study. Table S1. Language and literacy performance on tests used to classify outcomes at 8 years (Standard Scores). Table S2. Performance of the at‐risk normal outcome group relative to TD control and dyslexia group at t5. [file JCPP-61-672-s001.docx]

**Supporting information – Dyslexia and developmental language disorder: comorbid disorders with distinct effects on reading comprehension – by Snowling *et al*.**

**Appendix S1.** Measures used to classify groups at t1.

*Nonverbal ability*

Each child completed the Block Design and Object Assembly subtests from the Wechsler Scales of Intelligence (WPPSI-III; Wechsler, 2002) (Split-half reliability Block Design: = .84; Object Assembly = .85).

*Language*

*Comprehension*: At *t*1, *CELF Basic Concepts (CELF-Preschool 2 UK;* Wiig et al, 2006) was given. The child heard a sentence and had to select from a choice of three, the picture that represented the concept (α = .81-.87).

*Receptive Grammar*: *Sentence Structure* (*CELF-Preschool*, Wiig et al., 2006) was completed. The child heard sentences of increasingly complex syntactic structure and had to select from a choice of four pictures the one that conveyed the meaning of each (α = .83).

*Expressive Grammar*: the *Sentence Imitation Test* (*SIT-16*; Seeff-Gabriel, Chiat, and Roy, 2008) (α = .92) was completed. The child repeated sentences of increasingly complex syntactic structure. The score was number of sentences repeated correctly.

*Morphological Inflection*: The ability to produce grammatical inflections (third person / past tense) was assessed using the *Test of Grammatical Inflections* (*TEGI*, Rice and Wexler, 2001) (split-half *r* = .82 (past tense) and .92 (3^rd^ person singular))*.*

*Vocabulary*: The *CELF Expressive Vocabulary* test (*CELF-Preschool 2 UK*, Wiig et al., 2006 was given.

**Figure S1.** Participant Flow through Wellcome Language and Reading Study.
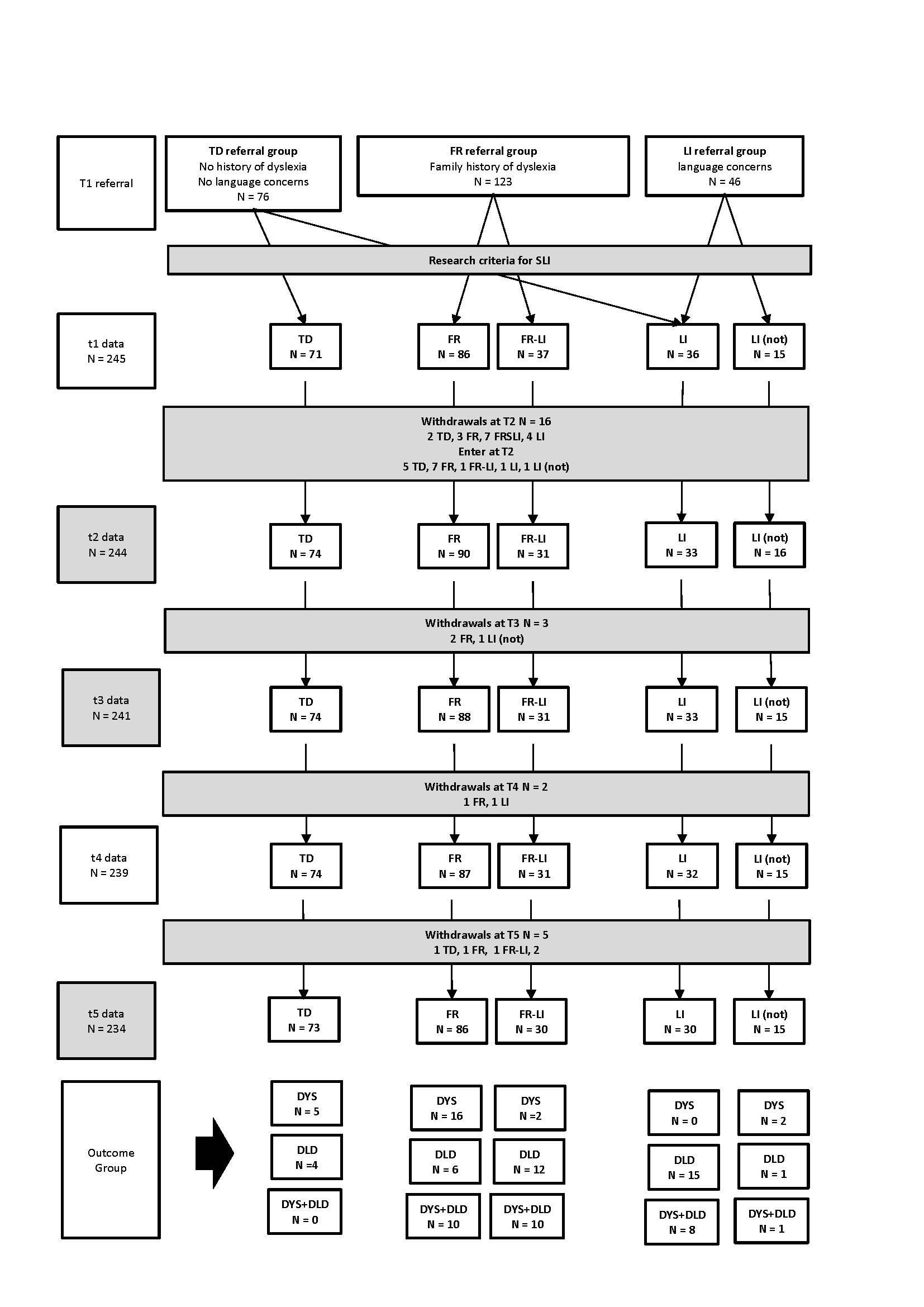


**Table S1.** Language and literacy performance on tests used to classify outcomes at 8 years (Standard Scores).

| **Test** | **TD control** |  | **Dyslexia** |  | **DLD** |  | **Dyslexia + DLD** |  |
| --- | --- | --- | --- | --- | --- | --- | --- | --- |
|  | Mean (SD) | Range | Mean (SD) | Range | Mean (SD) | Range | Mean (SD) | Range |
| Receptive Grammar  (TROG 2) | 105.05 (10.31) | 81-125 | 100.05 (13.83) | 69-118 | 75.21 (12.96) | 55-102 | 75.90 (13.89) | 55-106 |
| Recalling Sentences (CELF-4) | 111.06 (13.91) | 82-148 | 99.14  (15.21) | 58-130 | 83.10 (13.73) | 52-106 | 75.79  (13.93) | 52-100 |
| Expressive Vocabulary (CELF-4) | 117.67 (12.91) | 88-142 | 108.86 (11.16) | 94-130 | 92.11 (10.82) | 76-124 | 85.52 (11.51) | 64-106 |
| Reading (SWRT) | 110.21 (9.90) | 90-134 | 83.19 (7.53) | 69-94 | 104.24 (9.52) | 86-126 | 81.03 (8.22) | 69-95 |
| Spelling (WIAT-II) | 104.12  (11.87) | 78-140 | 79.52 (5.46) | 68-89 | 97.03  (9.39) | 76-119 | 79.07 (10.89) | 44-91 |

Table notes ^1^standard score

**Table S2.** Performance of the At-risk Normal Outcome Group relative to TD control and Dyslexia Group at t5.

|  | TD-control (N=64) |  | NR-risk (N=82) |  | DYS (N=21) |  |
| --- | --- | --- | --- | --- | --- | --- |
|  | Mean | SD | Mean | SD | Mean | SD |
| Vocab^1^ t5 | 0.52 | 0.63 | 0.34 | 0.55 | 0.13 | 0.59 |
| Vocab^1^ t6 | 0.46 | 0.71 | 0.28 | 0.61 | 0.10 | 0.77 |
| Nwrd^2^ t5 | 0.44 | 0.59 | 0.37 | 0.60 | -1.10 | 0.70 |
| Nwrd^2^ t6 | 0.44 | 0.66 | 0.38 | 0.60 | -1.10 | 0.69 |
| Read Comp^3^ t5 | 60.98 | 8.48 | 59.62 | 7.34 | 56.45 | 9.85 |
| Read  Comp^3^ t6 | 67.38 | 7.13 | 63.96 | 9.38 | 63.18 | 6.70 |

Table Notes: 1 Vocabulary Factor Score (expressive and receptive vocabulary); 2 Nonword Reading factor score (nonword reading accuracy and timed nonword reading efficiency); 3. York Assessment of Reading and Comprehension (YARC), Ability Score
